# Medicare Plan Switching Among Assisted Living Residents: The Role of Memory Care

**DOI:** 10.1093/geroni/igaf122.3506

**Published:** 2025-12-31

**Authors:** Wanru Liu, Gauri Gadkari, Jennifer Bunker, Kali Thomas, Eric Jutkowitz

**Affiliations:** Brown University, Providence, Rhode Island, United States; Brown University, Providence, Rhode Island, United States; Johns Hopkins University, Baltimore, Maryland, United States; Johns Hopkins University, Baltimore, Maryland, United States; University of Minnesota, Minneapolis, Minnesota, United States

## Abstract

Switching between Medicare Advantage (MA) and Fee-for-Service (FFS) commonly occurs after a health shock or residential relocation. However, little is known about how moving to an assisted living (AL) community influences enrollment decisions. This study examines Medicare plan switching among beneficiaries relocating to assisted living (AL) licensed for memory care versus general AL. Memory care ALs provide specialized dementia services but are approximately 36% more expensive than general ALs, potentially influencing Medicare choices. Using 2021 Medicare Beneficiary Summary Files and a national dataset of ALs, we tracked Medicare enrollment before and after moving to AL (N = 63,395) across 34 states licensing memory care ALs. A secondary analysis investigated switching among AL residents with dementia. Medicare plan switching varied based on AL licensing: FFS beneficiaries entering memory care-licensed ALs switched to MA less often than those entering general ALs (6.47% vs. 9.16%, p < 0.05). MA beneficiaries relocating to memory care compared to general AL were less likely to switch to another MA plan (10.95% vs. 13.08%, p < 0.05) or to FFS (4.31% vs. 5.55%, p < 0.05). Among individuals with dementia, residents with dementia in memory care ALs exhibited less FFS-to-MA switching compared to those in general ALs (3.40% vs. 4.76%, p < 0.05). These findings suggest that moving to an AL influences Medicare enrollment decisions; and, variation in switching exists between the type of AL (general vs memory care) and whether or not an individual has dementia.

